# Molecular markers dataset to assess the genetic diversity of oriental plane trees from historical sites in Lazio (central Italy)

**DOI:** 10.1016/j.dib.2022.108100

**Published:** 2022-03-25

**Authors:** Enrica Alicandri, Anna Maria Vettraino, Mariagrazia Agrimi, Mario Ciaffi, Elena Kuzminsky

**Affiliations:** Department for Innovation in Biological, Agro-food and Forest systems (DIBAF), University of Tuscia, Via S. Camillo de Lellis, snc I-01100, Viterbo, Italy

**Keywords:** *Platanus orientalis* L., Veteran trees, Gardens of XVI and XVII century, Genetic variation, Simple-sequence repeats, Inter Simple Sequence Repeat, Management and conservation of ancient plant material

## Abstract

The dataset presented in this article is related to the research paper titled “Dimensional and genetic characterization of the last oriental plane trees *(Platanus orientalis L.)* of historical sites in Lazio (central Italy)” (Ciaffi et al., 2022). Indeed, the molecular analyses reported in that article consisted in a comparison of Italian veteran plane trees with 12 certified accessions of *P. orientalis, P. occidentalis* and their hybrids *P. acerifolia* (4 individuals per species). First, LEAFY gene analyses allowed identifying 32 *P. orientalis* and two *P. acerifolia* in four sites of the province of Rome, confirming also that the two representative trees from the two gardens of the province of Viterbo belong to *P. orientalis*. Second, the use of Simple Sequence Repeat (SSR) and Inter Simple Sequence Repeat (ISSR) molecular markers provided useful information regarding the genetic relationships within and among all the historical sites. Owing to the use of SSR and ISSR molecular markers, a dataset of parameters related to the genetic diversity of the same plant material was obtained and presented in this article. For SSR markers, seven loci previously developed for *P. occidentalis* (Lang, 2010) and two specifically developed for *P. orientalis* (Rinaldi et al., 2019) were employed. For ISSR markers, DNA samples were amplified with eight primers before used for the determination of genetic stability of micro-propagated plantlets of *P. acerifolia* (Huang et al., 2009) and for the genetic characterization of plane trees within the formal gardens of Villa Lante of Bagnaia and Palazzo Farnese (Viterbo, Italy) (Ciaffi et al., 2018). To the best of our knowledge, this is the first report on the genetic diversity data for veteran oriental plane trees within heritage sites, which will offer helpful information for their management and conservation.

## Specifications Table

**Table:** 

Subject	Genetics
Specific subject area	Molecular markers analysis
Type of data	Table
How the data were acquired	For PCR amplification of both SSR and ISSR markers a Mastercycler Gradient Thermal Cycler 5331 was used. The SSR fragments were separated on ABI PRISM 3500 Genetic Analyzer and the amplicon signal peak height and size, from each allele, was quantified using ABI's Gene Mapper 4.0 software. The ISSR products were separated on 1.5% agarose gels and analyzed using the UVITEC Essential V6 Gel Imaging and Documentation. For both molecular markers the genetic parameters were obtained using the Power Marker 3.25 software.
Data format	Raw and analyzed data
Description of data collection	‐ Genomic DNA was extracted from leaves of 36 veteran plane trees from six historical sites of Lazio region and 12 representative accessions of *P. orientalis, P. occidentalis* and *P. acerifolia* used as references. ‐ 9 SSR primer pairs were used for the microsatellite amplification (plms29, plms113, plms176, 11FAM, PI2A, plms109, plms68, plms130 and plms147). ‐ 8 ISSR primers were used for the amplification (ISSR_12, ISSR_20, ISSR_24, ISSR_25, ISSR_13, ISSR_36, ISSR_14, ISSR_46).
Data source location	• City/Region: Grottaferrata, Lazio • Country: Italy • Latitude and longitude: 41° 47′ 11.64″ N, 12° 40′ 24.93″ E • City/Region: Frascati, Lazio • Country: Italy • Latitude and longitude: 41° 48′ 30.68″ N, 12° 40′ 33.98″ E • City/Region: Rome, Lazio • Country: Italy • Latitude and longitude: 41° 54′ 10.02″ N, 12° 29′ 46.92″ E • City/Region: Tivoli, Lazio • Country: Italy • Latitude and longitude: 41° 57′ 35.00″ N, 12° 47′ 53.00″ E • City/Region: Caprarola, Lazio • Country: Italy • Latitude and longitude: 42°19′44″N 12°14′12″E • City/Region: Viterbo (Bagnaia locality), Lazio • Country: Italy • Latitude and longitude: 42° 25′ 32.99″ N, 12° 9′ 16.99″ E
Data accessibility	Zenodo https://doi.org/10.5281/zenodo.6325140
Related research article	M. Ciaffi, A.M. Vettraino, E. Alicandri, A. Tomao, F. Adducci, E. Kuzminsky, M. Agrimi. Dimensional and genetic characterization of the last oriental plane trees *(Platanus orientalis* L.*)* of historical sites in Lazio (central Italy), Urban For. Urban Green. 69 (2022) 127,506. https://doi.org/10.1016/j.ufug.2022.127506

## Value of the Data

• This is the first report on the genetic diversity data for veteran oriental plane trees within different historical sites, which will offer helpful information for their management and conservation.
• The data can benefit to any researchers and/or professors working on the veteran plane trees germplasm to provide as much information as possible on the plant material origin as well as for the owners of historical sites, to individuate the better strategies for the tree heritage management.
• The SSR and ISSR markers data reported could be useful tools to provide a dataset for the comparison of the genetic diversity of veteran plane trees belonging to other historical sites of different localities.

## 1 Data Description

Among the nine selected SSR loci, one (plms147) was monomorphic, thus Table 1 reported the main genetic parameters of the eight remaining polymorphic loci. In the 48 plane specimens analyzed, the identified alleles for the codominant SSR markers were 71 in total, and the number of alleles ranged from 5 (pmls130 and pmls176) to 15 (PI2A), with an average of 8.88 alleles per locus (Table 1). H_e_ ranged from 0.348 (plms109) to 0.771 (11FAM) with a mean of 0.543, while H_o_ varied from 0.063 (plms68) to 0.833 (PI2A) with a mean of 0.339 (Table 1). The values of inbreeding coefficients (f) were all significantly different from zero with the exception of 11FAM and PI2A loci, with a mean of 0.450 (Table 1), indicating a relatively high level of inbreeding for the *Platanus* genotypes analyzed. The SSR markers differed in their ability to detect genetic variation among the 48 plane specimens analyzed, as shown by the differences in the number of alleles, genetic diversity (H_e_), major allele frequency (MAF) and polymorphism information content (PIC) (Table 1). Overall, the most informative SSR loci were 11FAM and PI2A, which showed the highest PIC (0.740 and 0.724, respectively) and H_e_ (0.771 and 0.757, respectively) values, and the lowest MAF values (0.333 and 0.344, respectively) (Table 1). On the other hand, the less informative SSR loci with the lowest PIC values were plms109 and plms68, which also were the ones showing the lowest and highest values for H_e_ and MAF parameters, respectively (Table 1).

**Table 1:** Genetic diversity parameters of SSR and ISSR markers used to genotype 48 plane tree specimens.

Markers	Allele No			MAF	H_e_	PIC	H_o_	f
**SSR loci**								
plms29	10			0.719	0.471	0.458	0.229	0.521
plms113	11			0.760	0.412	0.400	0.250	0.402
plms176	5			0.521	0.597	0.523	0.229	0.622
11FAM	11			0.333	0.771	0.740	0.813	−0.043
PI2A	15			0.344	0.757	0.724	0.833	−0.089
plms109	7			0.802	0.348	0.336	0.146	0.587
plms68	7			0.750	0.417	0.394	0.063	0.853
plms130	5			0.531	0.572	0.488	0.146	0.750
**Mean**	8.88			0.595	0.543	0.508	0.339	0.450
**St. Dev.**				0.189	0.160	0.150	0.305	0.347

**ISSR**	**Total bands**	**Polymorphic bands**	**Pol** **(%)**	**MAF**	**H_e_**	**PIC**		

ISSR_12	13	12	92%	0.832	0.259	0.216		
ISSR_20	10	8	80%	0.933	0.117	0.104		
ISSR_24	6	2	33%	0.882	0.154	0.123		
ISSR_25	10	8	80%	0.929	0.122	0.108		
ISSR_13	18	17	94%	0.826	0.257	0.213		
ISSR_36	14	13	93%	0.879	0.193	0.165		
ISSR_14	10	8	80%	0.921	0.134	0.118		
ISSR_46	11	10	91%	0.822	0.263	0.217		
**Mean**	11.5	9.75	80%	0.878	0.188	0.158		
**St. Dev.**				0.047	0.064	0.051		

Major Allele Frequency (MAF); Expected Heterozygosity (H_e_); Observed Heterozygosity (H_o_); Polymorphism Information Content (PIC); Inbreeding Coefficient (f); Polymorphism percentage (Pol %).

It is worth noting that the total number of alleles (71), the average number of alleles per locus (8.88), and the expected heterozygosity (0.543) detected here were higher than the corresponding values reported by Rinaldi et al. (2019) (62, 6.89, 0.406, respectively) [3], who analyzed 429 plane tree specimens belonging to 22 *P. orientalis* populations. In this context, however, it should be noted that, of the 34 private alleles identified, 23 were specific to the four accessions of *P. occidentalis* used as references (Table 2). Indeed, the same genetic parameters calculated only for the *P. orientalis* trees indicated that the total number of alleles was 36, with a range from 3 (plms176, plms109 and plms130) to 7 (11FAM, PI2A), and an average of 4.5 alleles per locus (Table 3). H_e_ ranged from 0.148 (plms109) to 0.651 (11FAM) with a mean of 0.352, while H_o_ varied from 0.026 (plms29, plms109 and plms130) to 0.868 (PI2A) with a mean of 0.325 (Table 3). Considering only *P. orientalis* trees, the most informative SSR locus was 11FAM, which showed the highest PIC (0.609) and H_e_ (0.651) values, and the lowest MAF values (0.526) (Table 3).

**Table 2:** Private SSR alleles identified in a single accession/specimen.

Species	Locus	Allele	Species	Locus	Allele
PorPB	11FAM	222	PocLAB	plms109	152
	PI2A	303		plms130	208
Por_SE2	plms113	210		plms131	214
	plms113	223		plms113	219
PorCRE_1	plms68	167		11FAM	227
	plms68	175		plms29	230
	PI2A	285		plms176	276
PorHAM	plms68	182		PI2A	276
	11FAM	208		PI2A	282

PacPB	plms113	205	PocM_11	plms29	206
	PI2A	286		plms113	216
PocILL_1	plms109	119		11FAM	229
	plms113	207		plms113	233
	11FAM	225		PI2A	280
	PI2A	278		PI2A	308
PocPB	plms29	222			
	plms29	226			
	PI2A	292			
	PI2A	299

**Table 3:** Genetic diversity parameters of SSR markers used for the 38 oriental plane trees.

Marker	Acronyms	Allele No	MAF	PIC	H_e_	H_o_
plms29	A1	5	0.868	0.234	0.241	0.026
plms113	B1	4	0.921	0.146	0.15	0.079
plms176	B2	3	0.605	0.449	0.528	0.079
11FAM	C1	7	0.526	0.609	0.651	0.816
PI2A	C2	7	0.763	0.392	0.406	0.868
plms109	D1	3	0.921	0.142	0.148	0.026
plms68	D2	4	0.895	0.189	0.195	0.079
plms130	D3	3	0.605	0.397	0.497	0.026
**Mean**		4.5	0.763	0.320	0.352	0.250
**St. Dev.**			0.162	0.168	0.194	0.343

Inbreeding level in plane tree genotypes where higher than in temperate wind pollinated species, which had a low level of inbreeding [6]. Indeed, the values of the observed heterozygosity (H_o_) were significantly lower than those of the expected ones (H_e_) for six of the eight SSR loci used (Table 1), indicating deviation from Hardy-Weinberg equilibrium and significant inbreeding, which is somehow unexpected for wind pollinated tree species such as *P. orientalis, P. acerifolia* and *P. occidentalis*, but confirming previous data obtained in *P. orientalis* from Rinaldi et al. [3].

Based on the ISSR analysis, 92 bands, ranging from 200bp to 2kb in size, were generated across the 48 plane specimens analyzed, with an average of 11.5 per primer (Table 1). The number of the bands varied from 6 (primer ISSR24) to 18 (primer ISSR13). Moreover, ISSR24 and ISS13 primers produced the lowest (2) and the highest (17) number of polymorphic bands, respectively (Table 1). Overall, 78 polymorphic bands were detected (80% polymorphism). H_e_ and PIC values for the dominant ISSR markers had relatively low and uniform values, with an average of 0.188 and 0.158, respectively (Table 1). In particular, H_e_ values ranged from 0.117 (ISSR20) to 0.263 (ISSR46), while PIC values were comprised between 0.104 (ISSR20) and 0.217 (ISSR46).

## 2 Experimental Design, Materials and Methods

Genomic DNA was extracted from leaves of the analyzed plane tree genotypes as described in [1]. A total of 36 veteran plane trees from historical sites of Lazio region and 12 representative *P. orientalis, P. occidentalis* and *P. acerifolia* accessions used as references were genotyped by using SSR and ISSR markers [1].

Nine SSR loci were used, of which seven previously developed for *P. occidentalis* [2] and two specifically for *P. orientalis* [3]. The forward primers of the nine SSR primer pairs were labeled with FAM, TAMRA, or JOE (Eurofins Genomics). PCR amplifications were performed in a 25 µL final volume containing 12.5 µL of 2X Go Taq Hot Start Colorless Master Mix (Promega Corporation, USA), 0.5 µM each of forward and reverse primers and 20 ng of genomic DNA, using the following thermal cycling conditions: 3 min initial denaturation step at 94 °C, followed by 30 cycles of amplification, each 1 min at 94 °C, 30 s at 52–60 °C, 40 s at 72 °C, and a final extension at 72 °C for 5 min. Amplification products were separated on ABI PRISM 3500 Genetic Analyzer (C.I.B.I.A.C.I. www.cibiaci.unifi.it). Allele sizes were determined using the ABI's Gene Mapper 4.0 software (Applied Biosystems) based on the GeneScan 500 LIZ size standard (Applied Biosystems). The raw data were entered in Microsoft EXCEL spreadsheet to create a codominant matrix.

For the ISSR markers, DNA samples were amplified with eight primers previously used for the determination of genetic stability of micro-propagated plantlets of *P. acerifolia* [4] and for the genetic characterization of plane trees within the formal garden of Villa Lante of Bagnaia (Viterbo, Italy) [5]. PCR reactions were carried out in a total volume of 25 μl containing 13 μl of GoTaq® Hot Start Colorless Master Mix (Promega Corporation, USA), 0.5 μM of each primer and 20 ng of genomic DNA. All reactions were performed using the following thermal cycling conditions: 4 min initial denaturation step at 94 °C, followed by 35 cycles of amplification, each 1 min at 94 °C, 1 min at 50–59 °C for 1 min, 2 min at 72 °C, and a final extension at 72 °C for 7 min. Every ISSR PCR reaction was independently conducted twice to verify amplification fidelity. ISSR fragments were separated on 1.5% (w/V) agarose gels, stained with ethidium bromide (0.001%), and visualized under UV light with the UVITEC Essential V6 Gel Imaging and Documentation System (Cleaver Scientific, Rugby, United Kingdom). In the analysis, only distinct, repeatable, and well-resolved fragments spanning from 200 to 2500bp were included. The DNA fragments (bands) in all genotypes were scored as present (1) or absent (0) for each of the ISSR markers, and the raw data were imported into a Microsoft EXCEL spreadsheet to build a binary matrix.

For SSR codominant markers each allele was reported according with its size (pb) and the genetic diversity per locus was evaluated by the following parameters: the number of alleles per locus (Allele No), Major Allele Frequency (MAF), expected (H_e_) and observed (H_o_) heterozygosity, Polymorphic Information Content (PIC) and inbreeding coefficient (f), using Power Marker 3.25 software [7].

The genetic diversity for each ISSR locus was evaluated by the following parameters: total number of bands obtained for each ISSR primer, number of polymorphic bands, percentage of polymorphism, MAF, H_e_ and PIC; with these latter three parameters determined using the Power Marker 3.25 software [7].

## Ethics Statements

No concern.

## CRediT authorship contribution statement

**Enrica Alicandri:** Methodology, Software, Investigation, Data curation, Formal analysis, Writing – review & editing. **Anna Maria Vettraino:** Writing – review & editing. **Mariagrazia Agrimi:** Funding acquisition, Writing – review & editing. **Mario Ciaffi:** Conceptualization, Supervision, Writing – original draft, Writing – review & editing. **Elena Kuzminsky:** Resources, Writing – review & editing.

## Declaration of Competing Interest

The authors declare that they have no known competing financial interests or personal relationships that could have appeared to influence the work reported in this paper.

## Data Availability

Tables S1 and S2 (Original data) (Zenodo).
